# Qualitative and Quantitative Analysis of the Major Constituents in Shexiang Tongxin Dropping Pill by HPLC-Q-TOF-MS/MS and UPLC-QqQ-MS/MS

**DOI:** 10.3390/molecules201018597

**Published:** 2015-10-14

**Authors:** Daxin Chen, Shan Lin, Wen Xu, Mingqing Huang, Jianfeng Chu, Fei Xiao, Jiumao Lin, Jun Peng

**Affiliations:** 1 Academy of Integrative Medicine, Fujian University of Traditional Chinese Medicine, Fuzhou 350122, Fujian, China; E-Mails: cdx1125@126.com (D.C.); lisa3350@163.com (S.L.); jianfengchu@126.com (J.C.); ybxwdj@hotmail.com (F.X.); jiumaolin@hotmail.com (J.L.); 2 Fujian Key Laboratory of Integrative Medicine on Geriatric, Fujian University of Traditional Chinese Medicine, Fuzhou 350122, Fujian, China; 3 College of Pharmacy, Fujian University of Traditional Chinese Medicine, Fuzhou 350122, Fujian, China; E-Mails: yaoxuexuwei@163.com (W.X.); hmq1115@126.com (M.H.)

**Keywords:** liquid chromatography, quadrupole time-of-flight tandem mass spectrometry, triple-quadrupole tandem, Shexiang Tongxin dropping pill

## Abstract

Shexiang Tongxin dropping pill (STP) is a traditional Chinese medicine formula that consists of total saponins of ginseng, synthetic *Calculus bovis*, bear gall, *Venenum bufonis*, borneol and *Salvia miltiorrhiza*. STP has been widely used in China and Southeast Asia for the treatment of cardiovascular diseases. In this study, a qualitative analytical method using high performance liquid chromatography coupled with quadrupole time-of-flight tandem mass spectrometry was developed for identification of the major constituents in STP. Based on the retention time and MS spectra, 41 components were identified by comparison with reference compounds and literature data. Moreover, using ultra-performance liquid chromatography coupled with triple-quadrupole tandem mass spectrometry in multiple-reaction monitoring mode, we quantified 13 of the identified constituents (ginsenoside Rg1, ginsenoside Rk3, cinobufagin, arenobufagin, bufalin, resibufogenin, tanshinone IIA, taurine, tauroursodeoxycholic acid, taurocholic acid, cholic acid, deoxycholic acid, and chenodeoxycholic acid). These results suggest that this new approach is applicable for the routine analysis and quality control of STP products and provides fundamental data for further *in vivo* pharmacokinetical studies.

## 1. Introduction

Traditional Chinese Medicine (TCM) typically involves the prescription of natural products in a composite formula, and has been widely used due to its specificity, effectiveness and low toxicity. There are a growing number of people who are using TCMs to achieve optimal health and prevent diseases [1,2,3,4,5]. Any prescribed formulas in TCM involve a complex system containing hundreds or even thousands of different chemical constituents. It is well accepted that the efficacy of TCM formulas is based on the synergistic effects of these components. Extensive efforts have been made to establish specific analytical methods in order to distinguish the chemical components in TCM prescriptions [6,7,8,9,10,11,12]. Shexiang Tongxin dropping pill (STP) is a Chinese FDA-approved formula that includes a combination of seven medicinal ingredients, including *moschus*, *Radix rhizoma ginseng*, *Calculus bovis*, bear gall, *Venenum bufonis*, borneol and *Salvia miltiorrhiza*. STP has been widely used in China and Southeast Asia for the clinical treatment of cardiovascular diseases. Previous pharmacological studies have proposed that STP can protect endothelial cells from atherosclerotic lesions by decreasing levels of endothelin-1 (ET-1), C reactive protein (CRP), tumor necrosis factor-α (TNF-α) and increasing levels of nitrogen oxide in the blood [13]. Moreover, STP can decrease levels of harmful lipids and improve abnormal hemorheology indices [14]. These therapeutic properties have been attributed to the major components in the prescription formula of STP [15,16,17,18,19,20,21]. 

Although the beneficial effects of STP have been well documented, the actual bioactive components of STP are still poorly understood. The constituents of each single herbal component in STP have been reported, however the chemical analysis of the final STP formula as a whole has not yet been elucidated [22,23,24,25,26,27,28]. This is necessary because the effects of TCMs do not simply involve the accumulated effects of individual components, but rather encompass the interactions between various components as a whole. In addition, changes and losses of certain constituents often occur during the production process of STP extraction, concentration and infusion, hence the contents of each component may also be changed. Therefore, qualitative and quantitative analysis of the total constituents in STP as a whole provides a more meaningful and specific outcome compared to the individual analysis of each component. In order to provide valuable information for quality control, as well as screening of the critical bioactive components, it is necessary to develop a specific and sensitive analytical method to identify and characterize the compounds in STP.

High performance liquid chromatography coupled with quadrupole time-of-flight tandem mass spectrometry (HPLC-Q-TOF-MS/MS) has become one of the most powerful analytical tools due to its high resolution and accurate mass measurements [29,30,31,32,33,34,35,36,37,38,39]. By comparing multiple-stage mass spectra with those of reference compounds and literatures, the unknown constituents can be identified. In addition, simultaneous quantitation of multi-components by ultra-performance liquid chromatography coupled with triple-quadrupole tandem mass spectrometry (UPLC-QqQ-MS/MS) has also been widely used in the analysis of TCM [40,41,42,43]. 

In this study, HPLC-ESI-Q-TOF method was established for separating and identifying the components in STP. All 41 components were identified based on their retention times and MS spectra data. Subsequently, UPLC-QqQ-MS/MS method was developed for the simultaneous quantitative analysis of 13 of the compounds in STP. This study represents the first detailed investigation into the components of STP and provides a valid method for its quality control and evaluation. 

## 2. Results and Discussion 

### 2.1. Optimization of HPLC Conditions

Chromatographic parameters were optimized to achieve a higher separation quality of the fingerprint chromatogram and a reduced analysis time. In order to obtain optimal chromatograms with good peaks and high sensitivity, different mobile phases including water/methanol and water/acetonitrile were examined. We determined that optimal separation of the analytes was achieved using water/acetonitrile as the mobile phase (Figure S1). The retention behavior of the compounds on reversed-phase HPLC columns was significantly affected by the pH of the mobile phase. Therefore we compared the effect of different acids, including formic acid, trifluoroacetic acid and phosphoric acid, as well as the amount of acid added into the mobile phase. We determined that the use of formic acid provided the greatest improvement during chromatographic separation, and also enhanced the formation of ions (Figure S2). The optimal conditions for a given factor was selected based on the observed peak capacity. The combination of the optimal factors and parameters, including chromatographic column, column temperature, mobile phase, flow rate and gradient elution program were analyzed in order to achieve the highest degree of separation for each peak (Figure S3 and Figure S4).

### 2.2. HPLC-Q-TOF-MS/MS Qualitative Analysis of Chemical Constituents in STP

**Figure 1:**
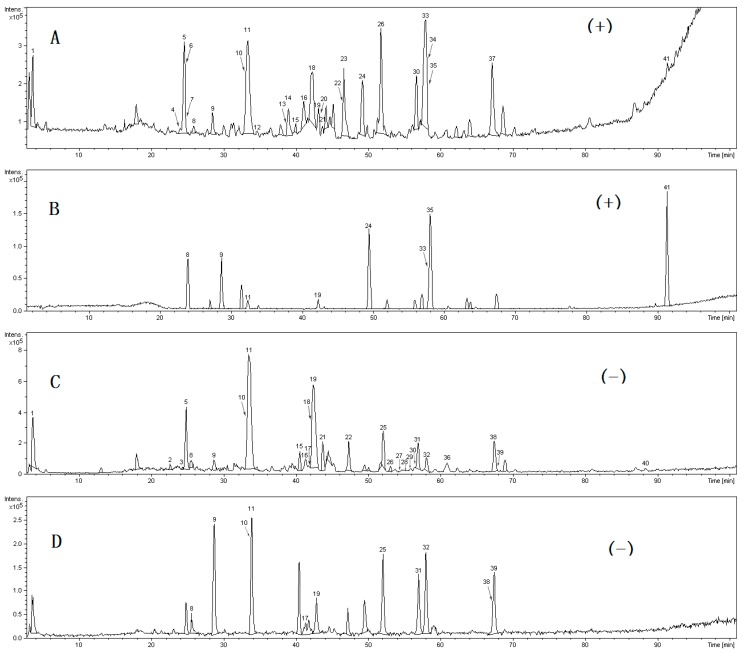
HPLC-Q-TOF-MS/MS total ion chromatogram (**A**) TIC of STP drug in positive mode (**B**) TIC of reference substances in positive mode (**C**) TIC of STP drug in negative mode (**D**) TIC of reference substances in negative mode. Standards are as follows: salvianolic acid B, arenobufagin, TDCA, TCA, Rg2, Rh1, bufalin, CA, UDCA, HDCA, resibufogenin, cinobufagin, CDCA, tanshinone IIA, and TDCANa.

**Figure 2:**
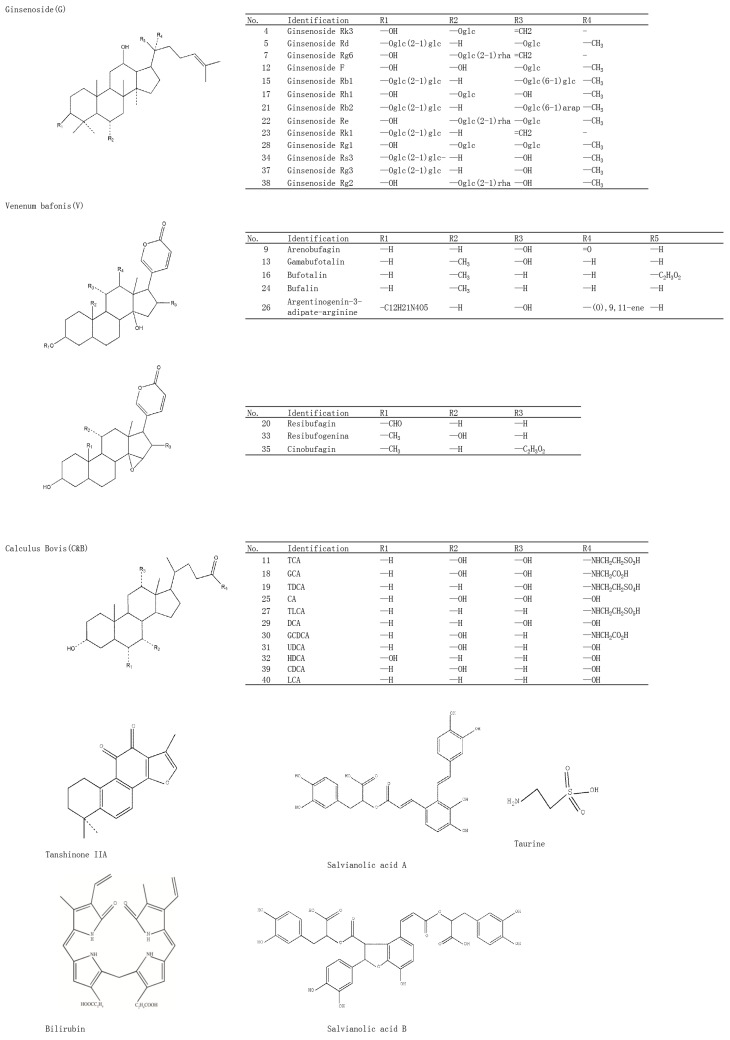
Chemical structures of the compounds identified in STP.

To characterize the chemical constituents in STP, HPLC-Q-TOF-MS/MS method was established. As shown in Table 1, a total of 41 components were initially identified or characterized, and 15 of those were identified unambiguously by comparing their retention times, mass accuracy, and fragmentation behaviors with data from the corresponding reference standards [37,39,44]. The remaining compounds were tentatively characterized based on MS data, and reference to available literature data. The mass error for molecular ions of all identified compounds was within ±9 ppm. The total ion chromatograms in positive and negative ion modes were displayed in Figure 1. All compounds were grouped into six types according to their structural characteristics, which included bufadienolides, triterpene saponins, tanshinones, salvianolic acids, bile acids and other types. The structures of all compounds are shown in Figure 2.

**Table 1:** Characterization of chemical constituents of STP by HPLC-Q-TOF-MS/MS.

No.	Source	Identification	Rt (min)	Formula	Negative Ion (*m*/*z*)			
					[M − H]^−^	Quasi-Molecular Ion	Error (ppm)	MSMS (*m*/*z*)
1	C & B	Taurine	3.7	C_2_H_7_NO_3_S	124.0063	124.0065	2	
2	R	Salvianolic acid A	23.8	C_26_H_22_O_10_	493.1129	493.1133	1	
3	C & B	Bilirubin	24.2	C_33_H_35_N_4_O_6_	583.2551	583.2518	6	583.2518, 469.3944,356.4968, 243.8168
4	G	Ginsenoside Rk3	NA	C_36_H_60_O_8_	619.4204	NA		
5	G	Ginsenoside Rd	24.9	C_48_H_82_O_18_	945.5417	945.5405	1	
6	R	Tanshinaldehyde	NA	C_19_H_16_O_4_	307.0965	NA		
7	G	Ginsenoside Rg6	NA	C_42_H_70_O_12_	765.4784	NA		
8 ^a^	R	Salvianolic acid B	25.5	C_36_H_30_O_16_	717.1450	717.1454	0	717.1454, 537.0995, 527.9935, 493.1069, 339.0521, 321.0405, 295.0633
9 ^a^	V	Arenobufagin	28.7	C_24_H_32_O_6_	415.2115	415.2115	0	415.2115, 397.2063, 371.2165, 353.2112, 277.1492, 196.5435, 151.0702
10 ^a^	C & B	TDCANa	33.8	C_26_H_44_NNaO_6_S	498.2884	498.2885	0	498.2907, 475.3216, 458.8943, 391.5463
11 ^a^	C & B	TCA	33.8	C_26_H_44_NO_7_S	514.2833	514.2856	4	514.2856, 496.2486, 482.3441, 124.1263
12	G	Ginsenoside Rf	NA	C_35_H_76_O_19_	799.4897	NA		
13	V	Gamabufotalin	NA	C_24_H_34_O_5_	401.2323	NA		
14	G	Notoginsenoside R2	NA	C_41_H_70_O_13_	769.4733	NA		
15	G	Ginsenoside Rb1	40.5	C_54_H_92_O_23_	1107.5946	1107.5927	2	1107.5927, 945.5389, 783.4901, 765.4580, 621.4343, 553.2887, 472.2575, 459.3803, 323.0972, 263.0742, 221.0678, 179.0538, 179.0538, 161.0395, 143.0272, 131.0323, 125.0221, 119.0351, 113.0260, 101.0208
16	V	Bufotalin	41.1	C_26_H_36_O_6_	443.2428	443.2452	5	443.2452, 407.2732, 380.9754, 375.2916, 256.8133, 203.8251, 167.9242, 138.0325
17 ^a^	G	Ginsenoside Rh1	41.8	C_37_H_64_O_11_	683.4403	683.4384	3	683.4384, 637.4330, 475.3704, 179.0470, 161.0398
18	C & B	GCA	42.6	C_26_H_43_NO_6_	464.3007	464.3015	2	
19 ^a^	C & B	TDCA	42.8	C_26_H_45_NO_6_S	498.2884	498.2907	5	498.2907, 464.2945, 451.3111, 391.1261, 321.1686, 201.2573
20	V	Resibufagin	NA	C_24_H_30_O_5_	397.2010	NA		
21	G	Ginsenoside Rb2	43.7	C_53_H_90_O_22_	1077.5840	1077.5795	4	1077.5795, 945.5318, 915.1144, 783.0026, 765.1212, 621.3652, 311.9821, 293.6514, 191.8123, 149.1853
22	G	Ginsenoside Re	47.2	C_48_H_82_O_18_	945.5417	945.5433	2	945.5433, 880.9436, 799.2672, 765.1661, 637.2542, 475.2767, 218.9817
23	G	Ginsenoside Rk1	NA	C_42_H_70_O_12_	765.4784	NA		
24 ^a^	V	Bufalin	NA	C_24_H_34_O_4_	385.2373	NA		
25 ^a^	C & B	CA	52.1	C_24_H_40_O_5_	407.2792	407.2811	5	407.2811, 389.2677, 363.2526, 325.6244, 289.2744, 233.8977, 215.3690, 205.9169
26	V	Argentinogenin-3-lutarate-arginine	53.6	C_38_H_58_N_4_0_8_	697.4171	697.4121	7	697.4121, 651.4153, 535.5406, 489.3649, 179.0546
27	C & B	TLCA	55.0	C_22_H_44_NO_10_	482.2960	482.2918	9	
28	G	Ginsenoside Rg1	55.3	C_43_H_72_O_14_	811.4838	811.4813	3	811.4813, 775.8988, 765.4577, 619.3911, 421.2024, 391.2774
29	C & B	DCA	55.8	C_24_H_40_O_4_	391.2843	391.2854	3	
30	C & B	GCDGA	56.2	C_26_H_43_NO_5_	448.3058	448.3065	2	448.3065, 409.2741, 391.2853, 389.2658, 365.3004
31 ^a^	C & B	UDCA	56.8	C_24_H_40_O_4_	391.2843	391.2848	1	409.2741, 391.2739, 373.8139, 354.4757, 152.9965
32 ^a^	C & B	HDCA	57.4	C_24_H_40_O_4_	391.2843	391.2851	2	409.2741, 391.2853, 389.2658, 118.1248
33 ^a^	V	Resibufogenin	NA	C_24_H_32_O_4_	383.2217	NA		
34	G	Ginsenoside Rs3	NA	C_44_H_74_O_14_	825.4995	NA		
35 ^a^	V	Cinobufagin	NA	C_26_H_34_O_6_	441.2272	NA		
36	G	Chikusetsusaponin Iva	61.6	C_42_H_66_O_14_	793.4374	793.4330	6	793.4330, 598.6542, 481.7612, 524.4589
37	G	Ginsenoside 20S-Rg3	NA	C_42_H_72_O_13_	783.4889	NA		
38 ^a^	G	ginsenoside Rg2	68.9	C_42_H_72_O_13_	783.4889	783.4873	2	783.4873, 765.1686, 617.0004, 409.2952, 391.2852, 313.1097
39 ^a^	C & B	CDCA	68.9	C_24_H_40_O_4_	391.2843	391.2853	3	391.2739, 345.4757, 329.1213, 97.0589
40	C & B	LCA	89.4	C_24_H_40_O_3_	375.2894	375.2888	2	
41 ^a^	R	Tanshinone IIA	NA	C_19_H_18_O_3_	293.1172	NA		
**No.**	**Source**	**Identification**	**Rt (min)**	**Formula**	**Positive Ion (*m*/*z*)**			
					**[M + H]^+^**	**Quasi-Molecular Ion**	**Error (ppm)**	**MSMS (*m*/*z*)**
1	C & B	Taurine	3.7	C_2_H_7_NO_3_S	126.0219	126.0222	2	126.0222, 115.3256, 108.8434, 97.2221, 91.7216
2	R	Salvianolic acid A	NA	C_26_H_22_O_10_	495.1286	NA		
3	C & B	Bilirubin	NA	C_33_H_35_N_4_O_6_	584.2629	NA		
4	G	Ginsenoside Rk3	24.5	C_36_H_60_O_8_	621.4361	621.4362	0	
5	G	Ginsenoside Rd	24.6	C_48_H_82_O_18_	947.5574	947.5501	8	
6	R	Tanshinaldehyde	24.6	C_19_H_16_O_4_	309.1121	309.1147	8	
7	G	Ginsenoside Rg6	24.6	C_42_H_70_O_12_	767.4940	767.4931	1	
8 ^a^	R	Salvianolic acid B	25.8	C_36_H_30_O_16_	719.1607	719.1613	1	719.1613, 323.0496, 295.0572, 181.0460
9 ^a^	V	Arenobufagin	28.4	C_24_H_32_O_6_	417.2272	417.2273	0	417.2273, 399.2188, 362.7495, 223.1056
10 ^a^	C & B	TDCANa	33.2	C_26_H_44_NNaO_6_S	500.4245	NA		
11 ^a^	C & B	TCA	33.5	C_26_H_44_NO_7_S	516.2990	516.2984	1	
12	G	Ginsenoside Rf	34.0	C_35_H_76_O_19_	801.5054	801.5075	3	
13	V	Gamabufotalin	38.9	C_24_H_34_O_5_	403.2251	403.2267	4	403.2267, 385.9531, 367.1296, 349.4632, 331.8123
14	G	Notoginsenoside R2	39.0	C_41_H_70_O_13_	771.4889	771.4861	4	
15	G	Ginsenoside Rb1	39.9	C_54_H_92_O_23_	1109.6102	1109.6030	6	1109.6030, 935.5613, 878.7854, 646.6751, 443.3798, 407.3652, 325.1114, 217.1888, 163.0566, 145.0507, 127.0384, 85.0294
16	V	Bufotalin	41.1	C_26_H_36_O_6_	445.2585	445.2585	0	
17 ^a^	G	Ginsenoside Rh1	NA	C_37_H_64_O_11_	685.4521	NA		
18	C & B	GCA	42.3	C_26_H_43_NO_6_	466.3163	466.3132	7	466.3132, 337.2523, 319.2353, 295.2026, 288.1602, 213.1621, 209.1339, 201.1658
19 ^a^	C & B	TDCA	42.8	C_26_H_45_NO_6_S	500.3040	500.3042	0	
20	V	Resibufagin	42.8	C_24_H_30_O_5_	399.2166	399.2158	2	399.2158, 387.2519, 297.7655, 223.1439, 211.1011, 145.0999, 131.0982, 179.0841, 105.0709, 91.0525
21	G	Ginsenoside Rb2	43.1	C_53_H_90_O_22_	1079.5997	1079.5942	5	
22	G	Ginsenoside Re	46.5	C_48_H_82_O_18_	947.5574	947.5510	7	
23	G	Ginsenoside Rk1	46.6	C_42_H_70_O_12_	767.4940	767.4931	1	
24 ^a^	V	Bufalin	49.1	C_24_H_34_O_4_	387.253	387.2527	1	387.2527, 370.9842, 352.9666, 340.2608, 255.2028
25 ^a^	C & B	CA	NA	C_24_H_40_O_5_	409.2949	NA		
26	V	Argentinogenin-3-lutarate-arginine	53.2	C_38_H_58_N_4_0_8_	699.4327	699.4299	4	699.4299, 681.4053, 598.7017, 582.3791, 331.2027, 278.1446, 250.1590, 175.1200, 157.1072
27	C & B	TLCA	NA	C_22_H_44_NO_10_	483.3038	NA		
28	G	Ginsenoside Rg1	NA	C_43_H_72_O_14_	813.4995	NA		
29	C & B	DCA	NA	C_24_H_40_O_4_	393.3000	NA		
30	C & B	GCDGA	56.1	C_26_H_43_NO_5_	450.3214	450.3204	2	450.3204, 415.3174, 321.2607, 278.7283, 215.1679, 175.1452, 161.1349, 147.1140, 107.0885
31 ^a^	C & B	UDCA	NA	C_24_H_40_O_4_	393.3000	NA		
32 ^a^	C & B	HDCA	NA	C_24_H_40_O_4_	393.3000	NA		
33 ^a^	V	Resibufogenin	57.7	C_24_H_32_O_4_	385.2373	385.2369	1	385.2369, 367.2214, 350.2243, 341.2244, 332.2025
34	G	Ginsenoside Rs3	57.8	C_44_H_74_O_14_	827.5151	827.5165	2	
35 ^a^	V	Cinobufagin	57.9	C_26_H_34_O_6_	443.2428	443.2425	1	443.2425, 401.2288, 385.2322, 367.2210, 349.2129, 187.1473, 151.0382
36	G	Chikusetsusaponin Iva	NA	C_42_H_66_O_14_	795.4525	NA		
37	G	Ginsenoside 20S-Rg3	68.6	C_42_H_72_O_13_	785.5046	785.5063	2	785.5063, 621.6871, 357.2785, 339.2647, 321.2458, 275.2027, 221.1526, 161.1345
38 ^a^	G	ginsenoside Rg2	NA	C_42_H_72_O_13_	785.5046	NA		
39 ^a^	C & B	CDCA	NA	C_24_H_40_O_4_	393.3000	NA		
40	C & B	LCA	NA	C_24_H_40_O_3_	377.3050	NA		
41 ^a^	R	Tanshinone IIA	92.3	C_19_H_18_O_3_	295.1269	295.1266	0	295.1266, 280.4501, 277.1173, 266.0878, 262.0933, 249.1212, 235.0725, 225.1178, 221.1251, 207.0771

G: Ginsenoside; V: *Venenum bufonis*; R: Red-rooted salvia; C: *Calculus bovis*; B: Bear gall; ^a^ Compared with a reference standard.

#### 2.2.1. Compounds from Ginseng

**Figure 3:**
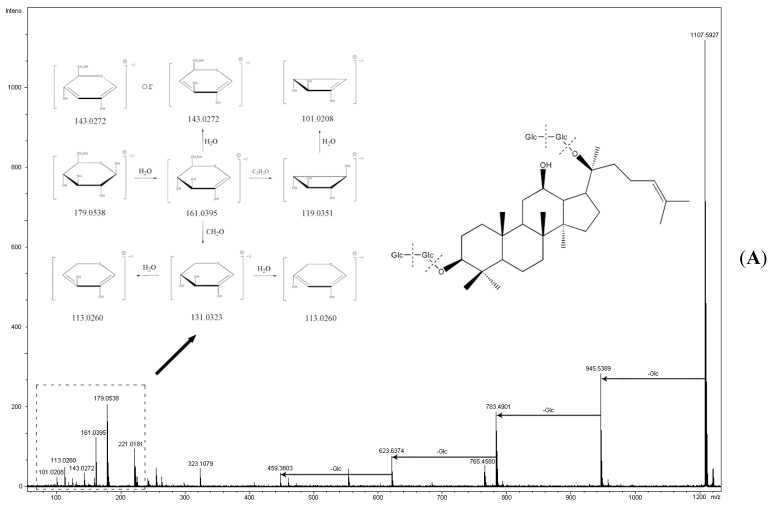

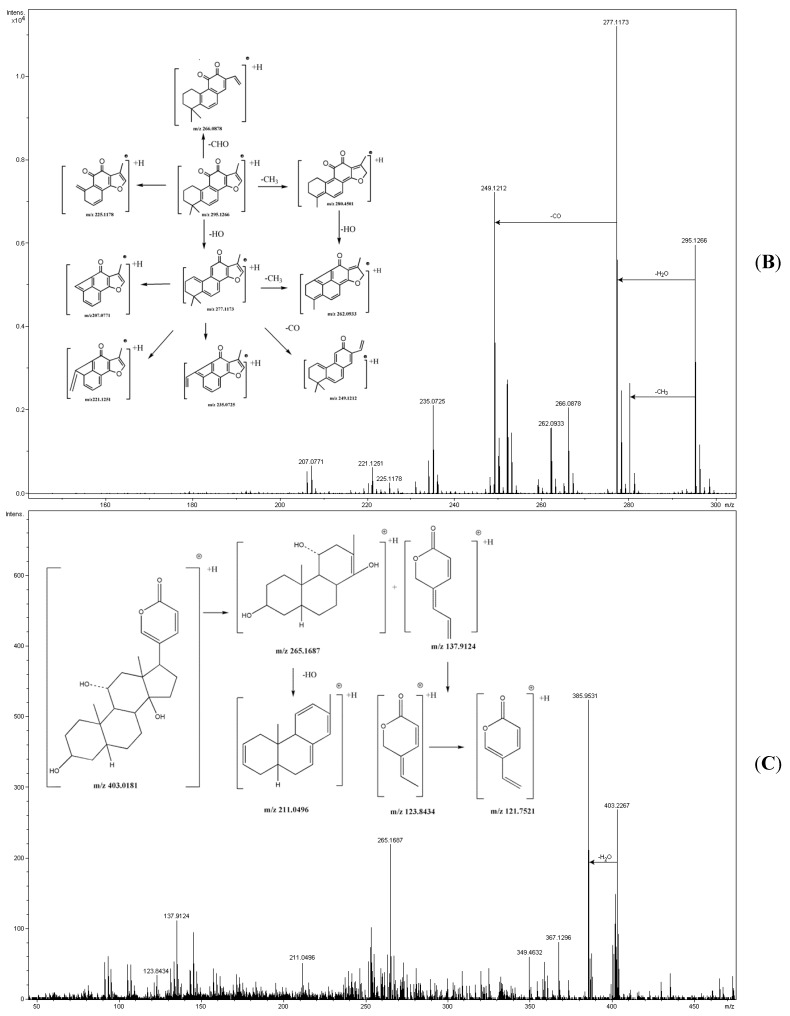

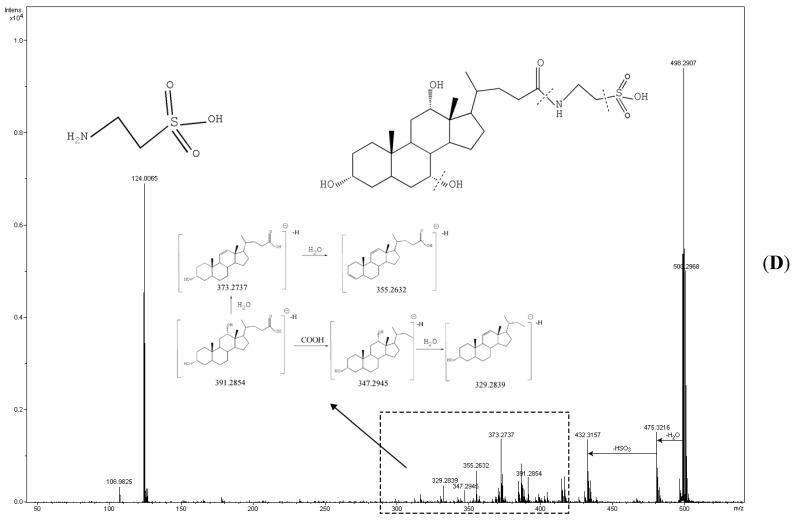
(**A**) The ESI-QTOF-MS spectra and the proposed fragmentation pathway of ginsenoside Rb1; (**B**) The ESI-QTOF-MS spectra and the proposed fragmentation pathway of tanshinone IIA; (**C**) The ESI-QTOF-MS spectra and the proposed fragmentation pathway of gamabufotalin; (**D**) The ESI-QTOF-MS spectra and the proposed fragmentation pathway of TDCA.

A total of 13 ginseng saponins were identified in this study, and these can be divided into those belonging to the protopanaxadiol (PPD) group, including ginsenoside Rd (Peak 5), ginsenoside Rf (Peak 12), ginsenoside Rg2 (Peak 38), ginsenoside Rk1 (Peak 23), ginsenoside Rs3 (Peak 34), and ginsenoside Rg3 (Peak 37); and the protopanaxatriol (PPT) group, including ginsenoside Rk3 (Peak 4), ginsenoside Re (Peak 22), ginsenoside Rb1 (Peak 15), ginsenoside Rb2 (Peak 21), ginsenoside Rh1 (Peak 17), ginsenoside Rg6 (Peak 7), and ginsenoside Rg1 (Peak 28). For saponin compounds, successive or simultaneous losses of sugar moieties are commonly produced in the negative MS/MS ion mode. In this study, ginsenoside Rb1 was used as an example to illustrate the fragmentation pathway of saponins as shown in Figure 3A. The precise molecular weight of ginsenoside Rb1 is 1107.5946 g/mol, and the main fragment ions including *m*/*z* 1107.5927 [M − H]^−^, 945.5389 [M − H-Glc]^−^, 783.4901 [M − H-2Glc]^−^, 765.4580 [M − H-(Glc-H_2_O)]^−^, and 459.3803 [C_32_H_56_O_3_]^−^ were observed via MS/MS screening. In addition, highly abundant ions at *m*/*z* 179.0538, 161.0395, 143.0272, 131.0323, 119.0351, 113.0260, and 101.0208 produced via cross fragmentation were also found.

#### 2.2.2. Compounds from *Salvia miltiorrhiza*

The main ingredients of *Salvia miltiorrhiza* were detected in this study, which included salvianolic acid A (Peak 2), salvianolic acid B (Peak 8), and tanshinone IIA (Peak 41). The main fragment ions of tanshinone IIA as analyzed by MS/MS screening were observed at *m*/*z* 295.1266 [M + H]^+^, 280.4501 [M + H-CH_3_]^+^, 225.1178 [M + H-CHO]^+^, 277.1173 [M + H-H_2_O]^+^, 262.0933[M + H-H_2_O-CH_3_]^+^, and 249.1212 [M + H-H_2_O-CO]^+^ in the positive ion spectrum (Figure 3B).

#### 2.2.3. Compounds from Toad Skin Secretion *(Venenum bufonis)*

Eight toad skin secretion compounds from *Venenum bufonis* were identified in this study. These compounds can be categorized into the *Venenum bufonis* I (VB I) group, which included arenobufagin (Peak 9), gamabufotalin (Peak 13), bufotalin (Peak 16), bufalin (Peak 24), and argentinogenin-3-adipate-arginine (Peak 26); and the *Venenum bufonis* II (VB II) group, including resibufagin (Peak 20), resibufogenin (Peak 33), and cinobufagin (Peak 35).

The mass fragmentation patterns were analyzed to verify the identification of these compounds. The results showed that the unconjugated bufosteroids can produce a series of neutral ions or positive ions containing rings A, B and C following the continuous loss of neutral H_2_O (18 Da) ion in the remaining steroidal rings. The main fragment ions of gamabufotalin include *m*/*z* 403.2267 as [M + H]^+^, 385.9531 [M + H-H_2_O]^+^, 367.1296 [M − H_2_O-CO]^+^, 349.4632 [M + H-C_2_OH_3_]^+^, and 265.1687 [M + H-C_8_H_9_O_2_]^+^ were observed in this study. The cleavage method of the bufosteroids without 16-hydroxyl substation showed obvious ion peaks of the lactonic ring E at *m*/*z* 121.7521 [C_7_H_5_O_2_]^+^ and *m*/*z* 137.9124 [C_8_H_8_O_2_ + H]^+^ in positive ion mode (Figure 3C).

#### 2.2.4. Compounds from *Calculus bovis* and Bear Gall

The major constituents of *Calculus bovis* and bear gall all have similar bile acid composition, and therefore we performed simultaneous analysis of these compounds. Eleven bile acids were identified, including taurine (Peak 1), bilirubin (Peak 3), TDCA (Peak 19), TCA (Peak 11), GCA (Peak 18), CA (Peak 25), UDCA (Peak 31), TLCA (Peak 27), GCDCA (Peak 30), HDCA (Peak 32), CDCA (Peak 39), DCA (Peak 29), and lithocholic acid (Peak 40).

In this study, four peaks (compounds 31, 32, 39, and 29) showed identical ions at *m*/*z* 391.2850 ± 4 ppm [M* − H]^−^ and *m*/*z* 783.4830 ± 3ppm [2M* − H]−. By comparison with reference standards, these four compounds were identified as UDCA (391.2848 [M* − H]^−^), HDCA (391.2851[M* − H]^−^), CDCA (391.2853 [M* − H]^−^), and DCA (391.2854[M* − H]^−^). The main fragment ions of compound 19 were observed at *m*/*z* 498.2907 [M − H]^−^, 475.3216 [M − H-H_2_O]^−^, 432.3157 [M − H-HSO_3_]^−^, and 391.2854 [M + H-H_2_O-C_2_H_7_N]^−^ in the negative ion spectrum. Furthermore, compound 19 showed the same fragmentation pathway to DCA at *m*/*z* 373.2737 [M* − H-H_2_O]^−^, *m*/*z* 355.2632 [M* − H-2H_2_O]^−^, *m/z* 347.2945 [M* − H-HCOO]^−^ and *m*/*z* 329.2839 [M* − H-H_2_O-HCOO]^−^ in the MS/MS spectrum, and therefore we deduced that compound 19 was TDCA (Figure 3D).

### 2.3. UPLC-QqQ-MS/MS Quantitative Analysis of Chemical Constituents from STP

**Figure 4:**
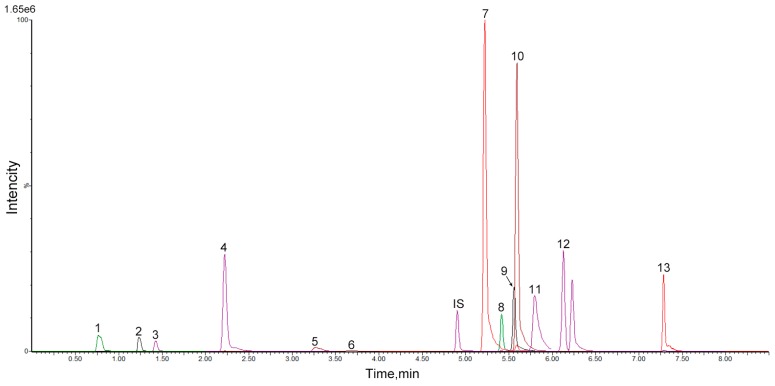
The MRM chromatograms of 13 markers and internal standard: (1) taurine, (2) ginsenoside Rg1, (3) arenobufagin, (4) TDCA, (5) TCA, (6) bufalin, (7) CA, (8) cinobufagin, (9) resibufogenin, (10) ginsenoside Rg3, (11) CDCA, (12) DCA, (13) tanshinone IIA, (IS) astragaloside*.*

The qualitative results indicated that ginsenoside Rg1, ginsenoside Rg3, cinobufagin, arenobufagin, bufalin, resibufogenin, tanshinone IIA, taurine, astragaloside, tauroursodeoxycholic acid, taurocholic acid, cholic acid, deoxycholic acid, and chenodeoxycholic acid were the major constituents in STP (Figure 4).

Furthermore, because of the similarity between their structures, retention time and ionization response in ESI-MS, astragaloside was chosen as the internal standard for the saponins, bile acids and tanshinones. In order to develop a sensitive and accurate quantitative method, the MS/MS fragmentation for each compound was investigated by direct infusion of the single standard solution into the mass spectrometer, and the resulting mass spectra of product ions were recorded. All chemical constituents were characterized according to their mass spectra in order to ascertain their precursor ions and to select proper product ions for MRM analysis. Finally, the most sensitive transition in MRM was selected.

### 2.4. Method Validation 

#### 2.4.1. Linearity and Detection Limit

The calibration curves plotted against the standard solution were constructed from the peak area ratios of each standard *vs.* concentrations of each constituent. Therefore we can determine that this method is sensitive for the quantitative determination of the major components in STP samples. The linearity of the calibration curves were verified by correlation study, where the correlation coefficients were better than 0.9990 within the test ranges. The LODs and LOQs for the 13 compounds were less than 0.51 and 1.70 μg/L, respectively (Table 2).

**Table 2:** Calibration curves, linear ranges, LOQ and LOD of 13 detected compounds (*n* = 3).

Compound	Linear	Linear Range (μg/mL)	r	LOQ (μg/mL)	LOD (μg/mL)
Taurine	y = 0.0707x − 0.0005	10.03–0.40	0.9999	0.1300	0.0390
Ginsenoside Rg1	y = 0.0574x − 0.0102	7.51–0.75	0.9998	0.6000	0.1800
Arenobufagin	y = 0.3438x − 0.0003	5.02–0.40	0.9996	0.0300	0.0090
TDCA	y = 0.0125x − 0.0061	25.28–3.75.	0.9992	1.7000	0.5100
TCA	y = 0.0525x − 0.0094	10.53–0.75	0.9996	0.6000	0.1800
Bufalin	y = 1.1663x + 0.0624	4.01–0.40	0.9999	0.0032	0.0010
CA	y = 0.0909x − 0.0211	10.07–1.01	0.9998	0.7800	0.2340
Cinobufagin	y = 0.1767x + 0.0523	5.02–0.75	0.9998	0.0003	0.0010
Resibufogenin	y = 0.9504x − 0.0596	4.01–0.50	0.9993	0.2100	0.0630
Ginsenoside Rg3	y = 0.048x + 0.0256	2.01–0.01	0.9994	0.0041	0.0012
CDCA	y = 0.2824x − 0.0241	5.01–0.75	0.9993	0.2900	0.0870
DCA	y = 0.2049x + 0.0145	4.01–0.40	0.9990	0.0050	0.0015
Tanshinone IIA	y = 34.5600x − 0.0030	0.05–0.01	0.9995	0.0042	0.0013

#### 2.4.2. Precision, Repeatability, Stability and Recovery

Precision, repeatability and stability of the method were also validated for each analyte. The RSDs were less than 4.88%, respectively. In conclusion, our experimental method provided high precision, repeatability, and stability. The accuracy of the method was determined by a recovery test (Table 3). As shown in Table 4, the recovery rate of the 13 standards varied from 98.24%–101.02% (RSD ≤ 4.48%). These results verified the high recovery and accuracy of this method.

**Table 3:** Precision, repeatability, stability of 13 detected components of STP.

Analyte	Repeatability		Precision RSD%		Stability
	Content (mg/g) ± SD	RSD% *n* = 6	Intra-Day *n* = 6	Inter-Day *n* = 9	RSD% *n* = 6
Taurine	35.2978 ± 0.7821	2.22	2.35	2.42	2.71
Ginsenoside RG1	8.7050 ± 0.2457	2.82	4.69	4.30	3.24
Arenobufagin	10.9163 ± 0.2826	2.59	3.13	3.95	4.33
TDCA	43.8546 ± 1.8145	4.14	3.96	3.08	2.47
TCA	29.7562 ± 1.3109	4.41	4.17	3.34	3.55
Bufalin	6.9432 ± 0.2124	3.06	3.88	4.20	3.40
CA	33.6313 ± 0.8419	2.50	3.52	3.58	2.58
Cinobufagin	19.1693 ± 0.4315	2.25	2.71	2.63	3.91
Resibufogenin	8.1053 ± 0.1211	1.49	2.88	3.00	3.00
Ginsenoside RG3	8.5907 ± 0.2642	3.08	2.63	2.60	2.25
CDCA	14.7924 ± 0.5840	3.95	4.79	3.46	2.12
DCA	5.7912 ± 0.1679	2.90	2.01	2.37	2.24
Tanshinone IIA	0.0208 ± 0.0007	3.54	4.00	4.55	4.88

**Table 4:** Recoveries of 13 detected components of STP.

Analyte	Original (μg)	Added (μg)	Detected (μg) (±SD *n* = 3)	Recovery (%) (±SD *n* = 9)	RSD (%)
Taurine	1764.89	2000.00	3734.82 ± 73.55	99.20 ± 3.00	3.02
		1500.00	3263.21 ± 55.01		
		1000.00	2756.97 ± 27.37		
Ginsenoside RG1	435.25	500.00	934.19 ± 22.09	98.86 ± 3.77	3.82
		400.00	826.68 ± 12.69		
		300.00	732.04 ± 14.85		
Arenobufagin	545.82	900.00	1422.16 ± 4.83	99.28 ± 3.49	3.52
		600.00	1156.98 ± 26.84		
		300.00	841.64 ± 10.52		
TDCA	2192.73	3000.00	5115.89 ± 43.01	98.64 ± 2.96	3.00
		2000.00	4195.34 ± 57.22		
		1000.00	3176.37 ± 43.66		
TCA	1487.81	2000.00	3435.51 ± 54.39	99.26 ± 3.31	3.34
		1500.00	2994.86 ± 70.89		
		1000.00	2487.12 ± 24.57		
Bufalin	347.16	500.00	843.70 ± 24.18	99.00 ± 4.18	4.23
		400.00	744.40 ± 18.15		
		300.00	642.24 ± 15.03		
CA	1681.57	2000.00	3682.31 ± 57.38	99.20 ± 3.09	3.11
		1500.00	3149.48 ± 52.01		
		1000.00	2678.33 ± 37.17		
Cinobufagin	958.47	1500.00	2428.88 ± 47.17	98.64 ± 3.38	3.00
		1000.00	1963.89 ± 36.52		
		500.00	1434.10 ± 3.47		
Resibufogenin	405.27	500.00	896.21 ± 13.03	98.24 ± 3.20	3.25
		400.00	795.04 ± 12.45		
		300.00	702.54 ± 14.18		
Ginsenoside RG3	429.54	500.00	928.54 ± 19.34	99.63 ± 3.55	3.56
		400.00	825.86 ± 17.65		
		300.00	729.51 ± 11.68		
CDCA	739.62	1500.00	2222.5 ± 54.47	98.67 ± 2.97	3.01
		750.00	1475.11 ± 17.65		
		500.00	1234.91 ± 19.78		
DCA	289.56	600.00	888.35 ± 23.94	99.38 ± 3.23	3.25
		300.00	584.70 ± 11.05		
		150.00	439.51 ± 4.75		
Tanshinone IIA	1.04	2.00	3.01 ± 0.09	101.02 ± 4.53	4.48
		1.00	2.05 ± 0.05		
		0.50	1.56 ± 0.03		

### 2.5. Sample Analysis

The established UPLC-QqQ-MS/MS analytical approach was subsequently used to determine the representative constituents in 8 batches of commercial STP products. The contents of the investigated compounds, based on their respective calibration curves are summarized in Table 3. There were significant variations among the contents of constituents between different batches of STP (Table 5). Among these, the main ingredients can be divided into bufadienolides, triterpene saponins, tanshinones, salvianolic acids, and bile acids. For instance, Sample 2 had the lowest contents of taurine, ginsenoside Rg1, and arenobufagin, whereas Sample 6 had the highest contents of CDCA, CA, cinobufagin, and resibufogenin. Moreover, the four bufadienolides, which includes arenobufagin, bufalin, cinobufagin, and resibufogenin, were found to be the major constituents, accounting for approximately 3.23%–14.06%, 2.75%–7.17%, 6.17%–19.17%, and 3.07%–8.11% composition of all batches, respectively. However, the contents of these four compounds varied significantly among the 13 different samples, which could result in the varying quality and efficacy of STP samples. Real sample data also demonstrated that UPLC-QqQ-MS/MS is a suitable method for the analysis of the active components in STP samples. Therefore, further studies of STP quality control should also focus on the composition of raw materials, as well as the optimization of parameters during processing and manufacturing.

**Table 5:** Contents of 13 analytes in different STP samples.

Lot No. Compounds (mg/g)	Sample 1	Sample 2	Sample 3	Sample 4	Sample 5	Sample 6	Sample 7	Sample 8
Taurine	13.8735	10.0161	15.1052	14.4826	13.6382	35.2978	24.4059	17.9399
Ginsenoside RG1	10.4003	4.7917	6.0041	5.4945	8.1709	8.7050	10.5062	9.8985
Arenobufagin	6.5724	3.2283	3.9044	6.4789	4.8138	10.9163	7.2296	14.0551
TDCA	43.2012	73.8723	52.7846	76.3674	96.1933	43.8546	78.2465	32.8261
TCA	21.2145	21.3171	33.0645	44.2204	52.5620	29.7562	47.4044	21.5164
Bufalin	4.3781	5.2646	2.7532	4.9919	3.4709	6.9432	5.4234	7.1698
CA	13.2086	25.3780	15.5592	15.1331	16.1522	33.6313	23.6953	23.8216
Cinobufagin	10.3166	6.1709	6.9125	11.3476	9.3716	19.1693	13.6489	15.7044
Resibufogenin	5.0181	3.0739	3.1118	5.4689	4.1013	8.1053	6.3188	7.4538
Ginsenoside RG3	17.1353	3.2188	8.0643	8.0672	13.4715	8.5907	13.3079	6.4563
CDCA	3.7939	4.6744	10.4556	6.6600	8.0945	14.7924	11.2672	5.8183
DCA	5.5619	3.6310	2.2421	5.1843	6.1205	5.7912	10.0939	9.4447
Tanshinone IIA	0.0026	0.0028	0.0207	0.0031	0.0134	0.0208	0.0239	0.0154

## 3. Experimental Section

### 3.1. Materials and Reagents

Reference substances of arenobufagin, bufalin, resibufogenin, cinobufagin, turo-ursodesoxycholic acid (TDCA), taurocholic acid (TCA), cholic acid (CA), chenodeoxycholic acid (CDCA), ursodesoxycholic acid (UDCA), and hyodeoxycholic acid (HDCA) were purchased from Must Bio-Technology Co., Ltd. (Chengdu, China). Tanshinone IIA, salvianolic acid B, gensenoside Rg2, and gensenoside Rh1 were purchased from the National Institute for the Control of Pharmaceutical and Biological Products (Beijing, China). The purity of each compound was determined to be higher than 98% by HPLC. LC-grade acetonitrile and formic acid were purchased from Merck (Darmstadt, Germany). Deionized water for HPLC analysis was purified using a Milli-Q system (Millipore, Billerica, MA, USA). Methanol for sample extraction was from Merck. STP raw materials were provided by the Inner Mongolia Conba Pharmaceutical Co., Ltd. (Inner Mongolia, China).

### 3.2. Preparation of Standard and Sample Solutions

Stock solutions of 13 standards (ginsenoside Rg1, ginsenoside Rk3, cinobufagin, arenobufagin, bufalin, resibufogenin, tanshinone IIA, taurine, tauroursodeoxycholic acid, taurocholic acid, cholic acid, deoxycholic acid, chenodeoxycholic acid and astragaloside as internal standard) were prepared individually by dissolving an accurately weighed amount of reference compound in methanol at concentrations of 1.397, 1.273, 0.975, 0.635, 1.272, 1.115, 0.167, 1.096, 0.885, 2.491, 1.119, 1.065, and 1.300 mg/mL, respectively. Next, aliquots of each stock solution were mixed, and diluted with methanol to achieve a series of standard working solutions for the construction of calibration curves.

In order to obtain the purest possible chemical constituents from STP extract, eight batches of STP samples were ground into fine powder without the capsule outer casing and thoroughly mixed. Next, 0.5 g powder was accurately weighed and extracted with methanol (50 mL) in an ultrasonic water bath (40 kHz, 500 W) for 30 min at room temperature. The samples were then centrifuged at 16,000× *g* for 10 min after replenishing methanol. The solution was incubated for 24 h at 4 °C and the resulting supernatant was used as the sample solution for LC/MS analysis. The resulting solution was filtered through a membrane with 0.22 µm pores prior to use. Sample injected volume was 6 µL.

### 3.3. Qualitative and Quantitative Analysis Conditions

#### 3.3.1. HPLC Method for Qualitative Analysis

HPLC-Q-TOF-MS/MS method for qualitative analysis was performed using a LC20AT HPLC instrument (Shimadzu, Kyoto, Japan) coupled with a micro-TOF-QII mass spectrometer (Bruker, Karlsruhe, Germany) equipped. The HPLC instrument includes a binary pump, an online degasser, and a thermostatically controlled column compartment. Chromatographic separation was carried out at 25 °C on an Inertsil ODS-SP C18 column (4.6 mm × 250 mm, 5 µm, GL Sciences Inc., Kyoto, Japan). The chromatographic conditions were as follows: flow rate of 0.8 mL/min, sample injection volume of 6 µL, mobile phase A (0.1% formic acid-water) and mobile phase B (100% acetonitrile) with a gradient elution program as follows: 0–5 min, 5%–5% B; 5–10 min, 5%–25% B; 10–60 min, 25%–48% B; 60–80 min, 48%–57% B; 80–100 min, 57%–90% B. Re-equilibration duration was 10 min between individual runs.

#### 3.3.2. Q-TOF Method for Qualitative Analysis

The micrOTOF-Q II mass spectrometer was equipped with electrospray ionization (ESI) source and operated in positive and negative mode. For the negative ion mode MS detection, the optimized operating parameters were as follows: capillary, +3500 V; end plate offset, +500 V; transfer time of 80 µs. For the positive ion mode, the optimized operating parameters were as follows: capillary, −4500 V; end plate offset, −500 V; transfer time of 120 μs. For both negative and positive ion mode, the same operation parameters were as follows: drying gas (N2) flow rate, 4.0 L/min; drying gas temperature, 180 °C; nebulizer, 2.0 Bar; hexapole Rf, 100.0 Vpp; quadrupole ion energy, 3.0 eV; collision Rf, 150.0 Vpp; prepulse storage time, 5μs. Argon was applied as the collision gas, The sample collision energy was set at 25–55 eV to obtain the fragment ions data. Acquisition and analysis of data was performed with Data Analysis Version 4.0 SP1 (Bruker Technologies). Each sample was analyzed in both positive and negative modes to provide abundant information for structural identification. Mass spectra were recorded across the range 50–1500 *m*/*z* with accurate mass measurement of all mass peaks. Accurate mass measurements of each peak from the total ion chromatogram (TIC) were obtained by means of an automated calibration delivery system using a dual-nebulizer ESI source that introduces a low flow (100 μL/min) of a calibrating solution (sodium formate solution, Bruker Technologies), which was performed daily before sample injection, and contains the internal reference masses at *m*/*z* 90.9766 and 1518.7125 in positive ion mode and *m*/*z* 112.9856 and 1472.7341 in negative ion mode. 

#### 3.3.3. UPLC-MS Method for Quantitative Analysis

UPLC-QqQ-MS/MS method was used for qualitative analysis, and a Waters (Milford, MA, USA) Acquity UPLC H-Class system coupled with Xevo TQD QqQ-MS with ESI was employed. Separations were accomplished on Waters CORTECS C18 (2.1 mm × 100 mm, 1.6 μm) at a flow rate of 0.25 mL/min. The column temperature was maintained at 45 °C. The mobile phase was acetonitrile (phase A) and water (containing 0.1% formic acid, phase B) with a gradient elution program as follows: 0–3 min, 30%–30% A; 3–5 min, 30%–70% A; 5–6 min, 70%–85% A; 6–7 min, 85%–85% A; 7–8 min, 85%–100% A, 8.01–10 min, 30%–30% A. The MS spectra were acquired in multiple reaction monitoring (MRM) mode. The collision gas was argon. The nebulizer gas and the heater gas both were nitrogen. The MS conditions were optimized as follows: capillary voltage 2500 V in positive ion mode; source temperature, 150 °C; dwell time, 20 ms, dry gas flow, 800 L/h, the temperature of dry gas, 200 °C. The most appropriate setting such as precursor ion, daughter ion, cone voltage, collision energy (CE) was adjusted according to each analyte (Table 6).

**Table 6:** The transitions and optimized MS parameters of 13 target markers and internal standard in the UPLC-QqQ-MS/MS analysis.

Compounds	Rt (min)	Precursor Ion (*m*/*z*)	Daughter Ion (*m*/*z*)	Con Voltage (V)	Collision Energy (eV)
Taurine	0.82	126.00	108.20	22	14
Ginsenoside Rg1	1.25	823.30	643.50	80	40
Arenobufagin	2.23	417.20	399.10	45	30
TDCA	3.39	522.28	486.61	50	25
TCA	3.41	538.00	538.00	60	2
Astragaloside	4.91	807.00	807.00	80	30
Bufalin	5.22	387.30	351.20	35	22
CA	5.41	391.30	355.30	0	20
Cinobufagin	5.56	443.50	365.20	60	20
Resibufogenin	5.59	385.30	367.20	35	18
Ginsenoside Rg3	5.75	807.00	365.00	90	5
CDCA	6.13	357.60	161.20	40	20
DCA	6.23	357.60	161.20	40	20
Tanshinone IIA	7.28	295.10	277.00	40	20

### 3.4. Validation of Quantitative Method

#### 3.4.1. Calibration Curve, LOD, and LOQ

For the calibration curves, at least six concentrations of calibration standard solution were made and analyzed in triplicate. Next, the calibration curve of each analyte was constructed by plotting the peak area *vs.* the corresponding concentration. 

The mixed standard solution with the lowest concentration was further diluted to a certain concentration to evaluate the LODs and LOQs. The LODs and LOQs were determined at an S/N ratio of 3 and 10, respectively.

#### 3.4.2. Precision, Stability, Repeatability, and Recovery

The analysis of intra- and inter-day precisions was carried out by six repetitive injection of a mixed standard solution, in the same day and once a day for three consecutive days, respectively. Both assays were determined by performing three different concentration levels of the standards. Six samples were prepared independently to verify the repeatability of the experiment. To investigate the stability of the samples, each sample solution was analyzed every 4 h within 24 h, and stored at room temperature. The recovery was used to evaluate the accuracy of the method, and was determined by adding the mixed standard solutions at three different concentration levels (low, medium, and high) to 0.10 g of the known amounts of STP sample (Sample 6). The mixture was then extracted and analyzed. Three replicates were performed at each level. The percentages of recovery were calculated according to the following equation: (found amount − original amount) × 100%/spiked amount. The RSD was used to evaluate the results.

## 4. Conclusions

Our study demonstrates for the first time, the chemical profile of STP through a thorough and systematic investigation using HPLC-Q-TOF-MS/MS. In this study, a rapid and sensitive method based on HPLC-Q-TOF-MS/MS and UPLC-QqQ-MS/MS was established for the separation, identification and determination of the chemical constituents of STP. We identified a total of 41 components, including triterpene saponins, bufadienolides, bile acids, phenylally compounds and other compounds, which were successfully separated and identified by HPLC-Q-TOF-MS/MS. We also established the optimized UPLC-QqQ-MS/MS method for simultaneous determination of 13 compounds in STP under optimized UPLC conditions. The MRM mode of QqQ-MS/MS using our method enabled identification of target compounds with high sensitivity even at low concentrations, through comparison with standards. Rapid analysis performed within 10 min also facilitated the efficient quantitation of the target compounds in STP. This analytical method also verified its efficiency through method validation (linearity, precision and recovery test). Consequently, our method provided an accurate and efficient tool for the quality control of STP. The results of this study will aid in the discovery and analysis of biologically active compounds in STP, as well as facilitating improvements in the quality control standard of this commonly used multi-component TCM formula.

## Supplementary Materials

Supplementary materials can be accessed at: http://www.mdpi.com/1420-3049/20/10/18597/s1.
