# Supplementary material for: Qualitative and Quantitative Analysis of the Major Constituents in Shexiang Tongxin Dropping Pill by HPLC-Q-TOF-MS/MS and UPLC-QqQ-MS/MS
Source: Molecules. 2015 Oct 14;20(10):18597–619. doi: 10.3390/molecules201018597 (PMC6331871; doi:10.3390/molecules201018597)
Supplement: Supplementary file 1 [file molecules-20-18597-s001.pdf]

Supplementary Materials

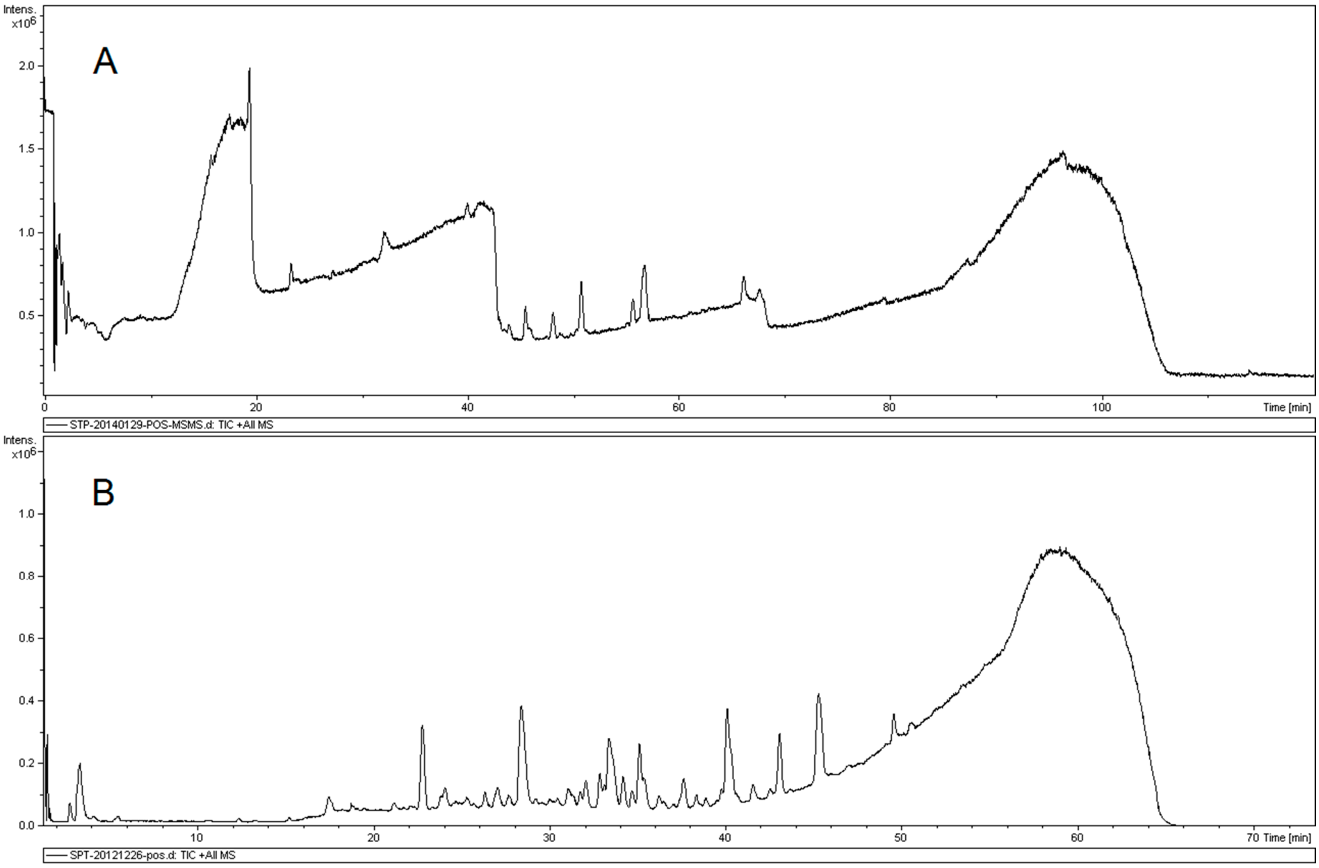

Figure S1. The HPLC-Q-TOF-MS/MS total ion chromatogram of STP by different mobile phases. (**A**) TIC of water/methanol as the mobile phases; (**B**) TIC of water/acetonitrileas the mobile phases.

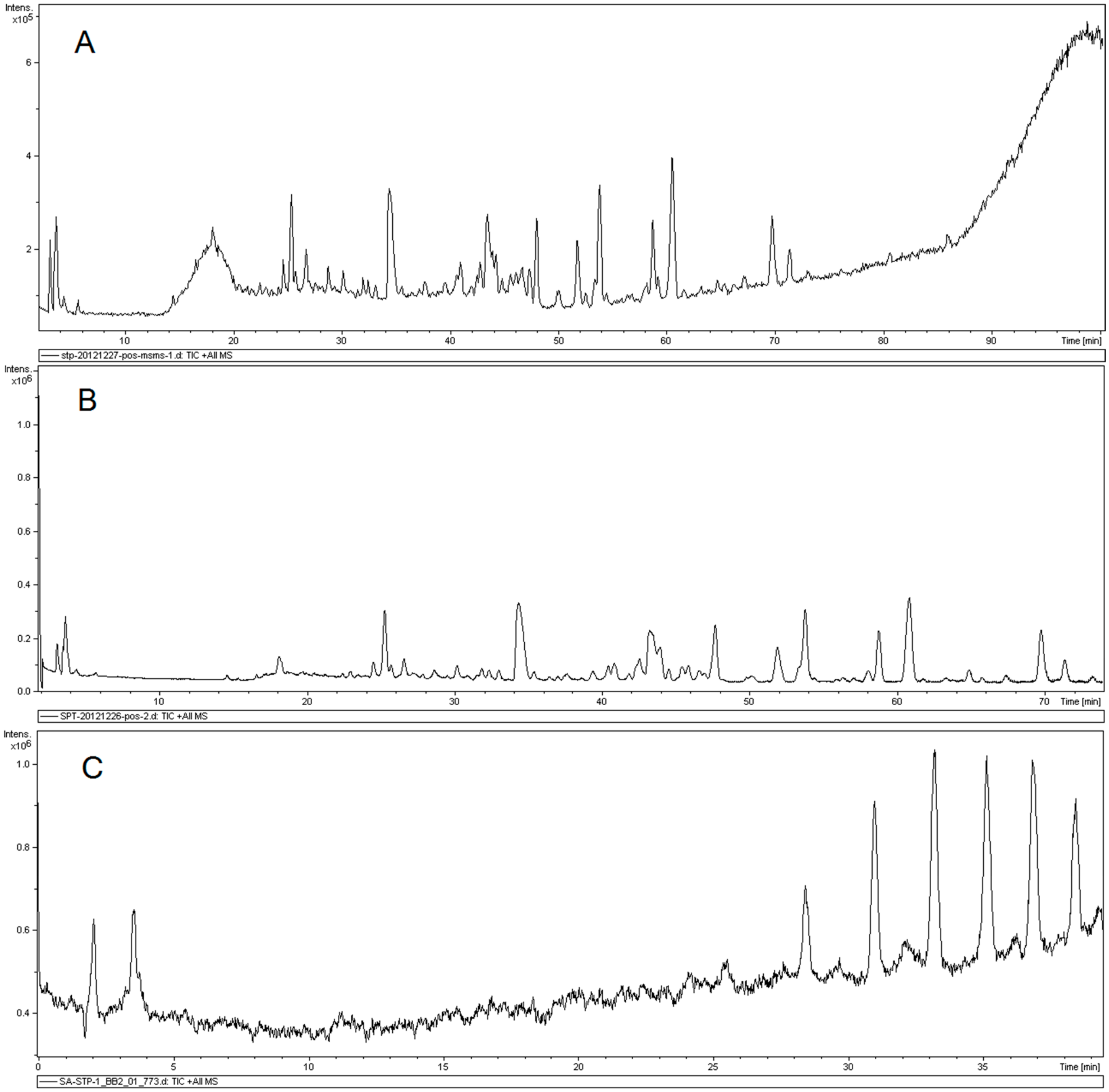

Figure S2. The HPLC-Q-TOF-MS/MS total ion chromatogram of STP by different acids in the mobile phases. (**A**) TIC of formic acidin the mobile phases; (**B**) TIC of trifluoroacetic acid in the mobile phases; (**C**) TIC of phosphoric acid in the mobile phases.

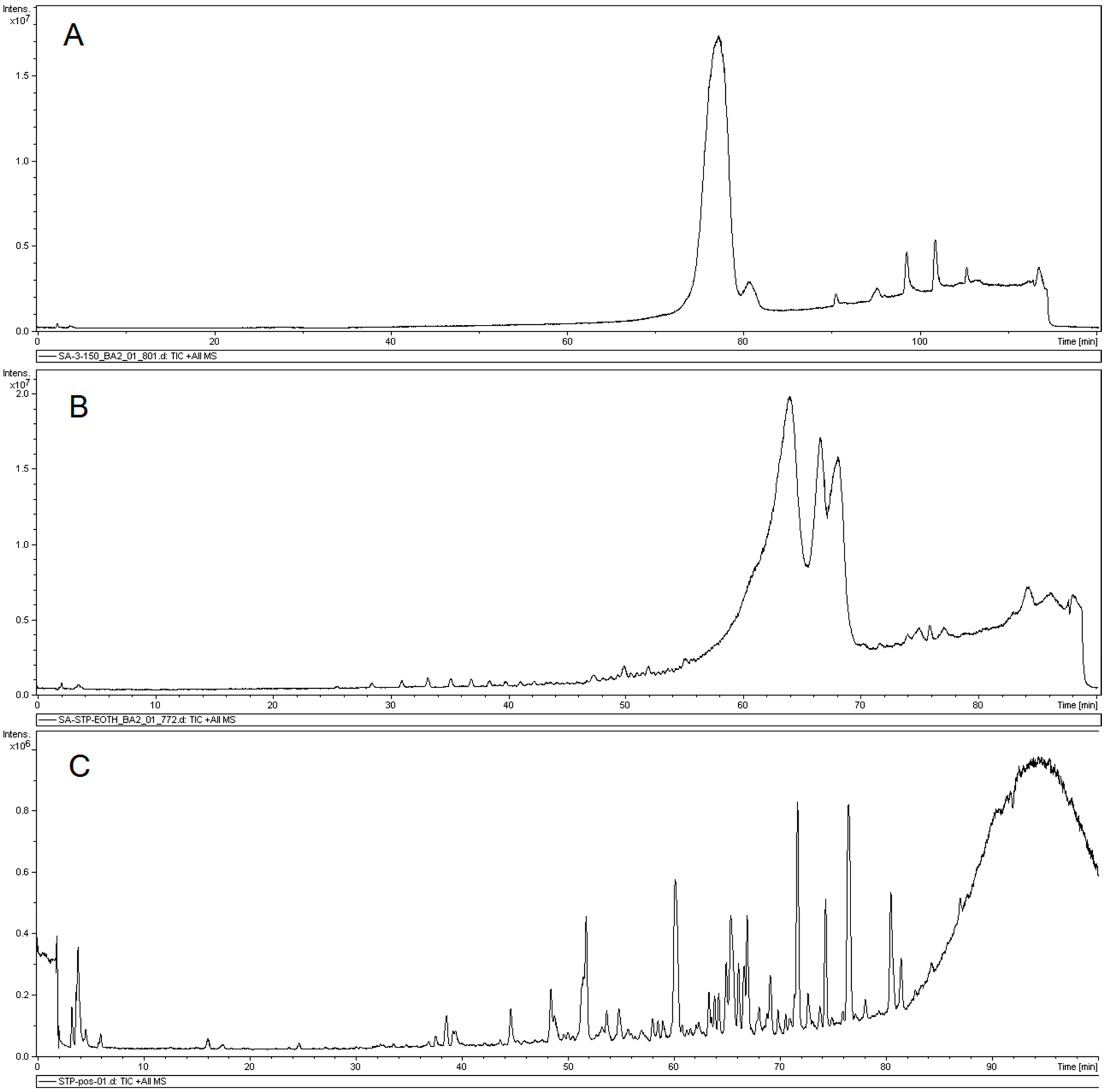

Figure S3. The HPLC-Q-TOF-MS/MS total ion chromatogram of STP by different chromatographic columns. (**A**) TIC of Agilent C18; (**B**) TIC of Ultimate XB ODS-C18 column; (**C**) TIC of Inertsil ODS-SP C18 column.

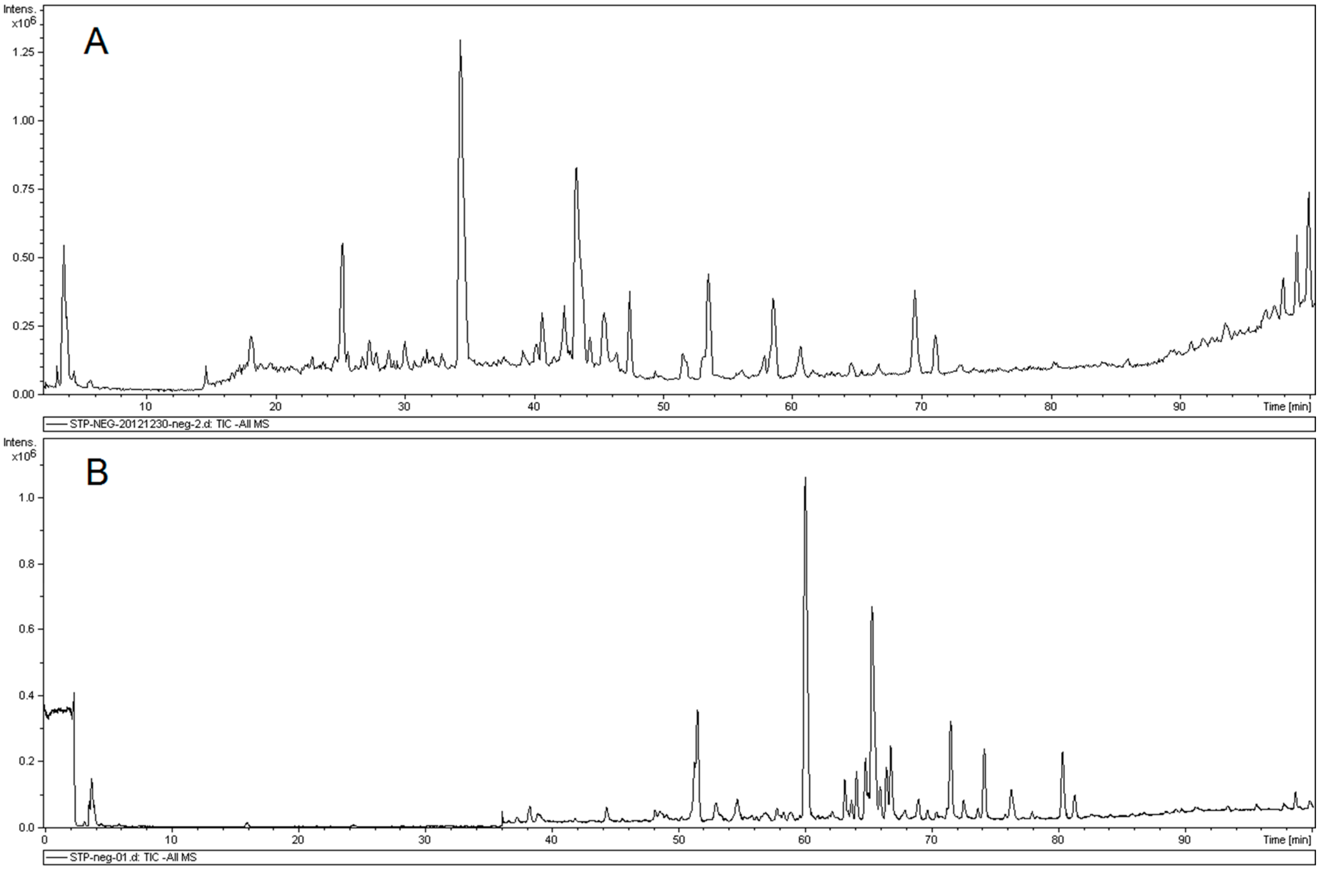

Figure S4. The HPLC-Q-TOF-MS/MS total ion chromatogram of STP by different programs of gradient elution in negative model. Both (**A**) and (**B**) are the different programs which had been tried.
